# The systematic analysis of coding and long non-coding RNAs in the sub-chronic and chronic stages of spinal cord injury

**DOI:** 10.1038/srep41008

**Published:** 2017-01-20

**Authors:** Raquel Cuevas-Diaz Duran, Han Yan, Yiyan Zheng, Xingfan Huang, Raymond Grill, Dong H. Kim, Qilin Cao, Jia Qian Wu

**Affiliations:** 1 The Vivian L. Smith Department of Neurosurgery, McGovern Medical School, The University of Texas Health Science Center at Houston, Houston, TX 77030, USA; 2 Center for Stem Cell and Regenerative Medicine, UT Brown Foundation Institute of Molecular Medicine, Houston, TX 77030, USA; 3 Department of Bioengineering, Rice University, Houston, TX 77005, USA; 4 Department of Integrative Biology and Pharmacology, McGovern Medical School, The University of Texas Health Science Center at Houston, Houston, TX 77030, USA

## Abstract

Spinal cord injury (SCI) remains one of the most debilitating neurological disorders and the majority of SCI patients are in the chronic phase. Previous studies of SCI have usually focused on few genes and pathways at a time. In particular, the biological roles of long non-coding RNAs (lncRNAs) have never been characterized in SCI. Our study is the first to comprehensively investigate alterations in the expression of both coding and long non-coding genes in the sub-chronic and chronic stages of SCI using RNA-Sequencing. Through pathway analysis and network construction, the functions of differentially expressed genes were analyzed systematically. Furthermore, we predicted the potential regulatory function of non-coding transcripts, revealed enriched motifs of transcription factors in the upstream regulatory regions of differentially expressed lncRNAs, and identified differentially expressed lncRNAs homologous to human genomic regions which contain single-nucleotide polymorphisms associated with diseases. Overall, these results revealed critical pathways and networks that exhibit sustained alterations at the sub-chronic and chronic stages of SCI, highlighting the temporal regulation of pathological processes including astrogliosis. This study also provided an unprecedented resource and a new catalogue of lncRNAs potentially involved in the regulation and progression of SCI.

Spinal cord injury (SCI) is one of the most debilitating neurological diseases. In the United States, SCI affects more than 300,000 people, and approximately 11,000 new SCI cases occur every year^1^. The majority of SCI patients are in the chronic phase of SCI because of the lack of cure^2^. Despite the efforts devoted to treatment and patient care, there are still no effective therapeutic solutions for SCI. Understanding the underlying cellular and molecular mechanisms, and particularly the regulation of SCI pathophysiological events in a systemic manner, is critical for developing promising treatment strategies.

So far, few studies have attempted to understand the altered expression of genes related to SCI at a global level and most of these studies have used microarrays^3^,^4^. Compared to microarrays, RNA-Sequencing (RNA-Seq) possesses several advantages, such as a larger dynamic range of detection, higher sensitivity and specificity, and an enhanced ability to interrogate any location in the genome^5^. Previous work in our lab has demonstrated the power of RNA-Seq technology in characterizing the transcriptomic alterations in mouse contusive SCI models via integrated transcriptomic and network analyses, which revealed new pathways and candidate molecular targets for acute and sub-acute SCI^6^. In this study, we investigated the molecular mechanisms of the sub-chronic and chronic SCI in rat models by examining the changes in expression of both protein-coding and long non-coding genes at 1 month (1 M), 3 months (3 M), and 6 months (6 M) after injury, respectively. Our results demonstrated that a high level of transcriptional disturbance persists during the sub-chronic and chronic injury phases, with many genes enriched in pathways such as immune and inflammatory responses, as well as gliosis.

Genome-wide analyses have indicated that non-coding RNAs comprise a major part of the genome and revealed another essential dimension of gene regulation^7^. A large number of lncRNAs (over 200 nt in length) have been discovered in recent years and been shown to play critical roles in various biological processes including central nervous system development^8^,^9^ and diseases^10^,^11^. Rat lncRNAs have never been studied in SCI. In the current study, we thoroughly annotated the characteristics of lncRNAs in the rat genome, and predicted the potential regulatory function of these non-coding transcripts by correlating the differential expression patterns of lncRNAs with those of protein-coding genes. Further, we searched for transcription factor (TF) motifs enriched in the upstream regulatory regions of these differentially expressed (DE) lncRNAs, and identified DE lncRNAs that are homologous to human genomic regions which contain single-nucleotide polymorphisms (SNPs) associated with neurological diseases.

In summary, this is the first comprehensive study using RNA-Seq to analyze the transcriptomic alterations of both coding and long non-coding genes in the sub-chronic and chronic stages of SCI. It provided a new perspective for the SCI field and a catalogue of lncRNAs potentially involved in the regulation and progression of this disorder.

## Results

### Overview of the rat transcriptome

**Figure 1:**
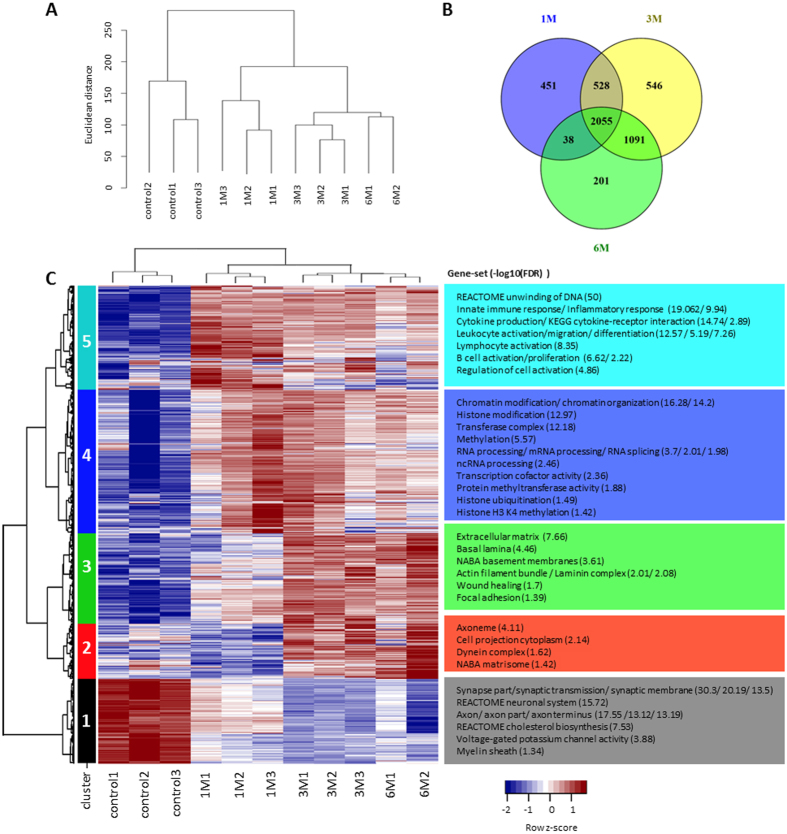
Gene expression profiles at 1 M, 3 M, and 6 M post-SCI compared to sham controls. (**A**) Consensus dendrogram of 30,443 annotated genes, organized by Euclidean distance between log2-transformed quantile-normalized FPKM values. (**B**) Venn diagram depicting the extent of overlap between time points. (**C**) Hierarchical cluster analysis displaying temporal gene expression patterns of DEGs for each sample. Highly enriched gene-sets and ontologies were identified (hypergeometric test FDR < 0.05). The −log10(FDR) is indicated in parenthesis. The row z-scores depict the expression values normalized by gene.

We mapped the sequenced reads to the Ensembl rat reference genome and transcriptome (Rnor6). Due to a lack of a comprehensive rat lncRNAs database, we combined the known and predicted lncRNA annotations from Ensembl and NCBI. On average, samples had 33.6 million reads with a standard deviation of 7.7 million reads (see Supplementary Table S1 for a detailed description of the number of reads, mapping rate, and processing batch for all samples). Approximately 91.1% of all fragments were mapped to the rat reference genome. Pairwise Pearson correlation coefficients among all samples were calculated based on the log2 transformed and quantile normalized FPKM (Fragments Per Kilobase of transcript per Million mapped reads) value of 30,443 genes. The average correlation coefficient for pairwise comparisons of replicates at each time point was 0.984 with a standard error of 0.005, indicating a high level of consistency among biological replicates. The Pearson correlation matrix for all samples is included in Supplementary Table S1. Based on Euclidean distance of transformed FPKM values, a consensus dendrogram was constructed (Fig. 1A). The most discriminant tree branches represent control and SCI samples; Within the SCI samples, 3 M and 6 M had the highest similarity. Using the Washington University Epigenome Browser^12^, we set up an interactive, searchable database to display gene expression levels of SCI samples at all time points and made it freely available to the research community. Protein-coding genes and lncRNAs are exhibited as separate tracks (http://jiaqianwulab.org/ratSCI/ratSCI.html).

Gene expression levels (FPKM) and raw count data matrices were generated for downstream analyses (Supplementary Table S2). A gene was considered expressed if the FPKM > 1 in at least one of the samples. This filtering process yielded 13,847 expressed protein-coding genes and 1,035 expressed lncRNA genes.

### Transcriptomic dynamics in the sub-chronic and chronic phases of SCI

Analysis of differential gene expression was performed by comparing the expression of all genes at each time point, both protein-coding and long non-coding, to that of the control group. The numbers of DE genes (DEGs) in each category are summarized in Table 1. A list of DE protein-coding (4,633) and lncRNA (277) genes from all time points was compiled and used for further analysis. A complete list of DEGs at each time point and those that either overlap among all three time points or are unique to one time point is provided in Supplementary Table S3. Expression profiles of genes that code for selected specific cell markers^13^,^14^,^15^ are available in Supplementary Table S4.

Enriched canonical pathways at each time point generated by using Ingenuity Pathway Analysis (IPA), including gene members for each pathway, are available in Supplementary Table S5. As shown in the Venn diagram (Fig. 1B), 2,055 DEGs overlapped among all three time points. The most enriched pathways throughout the sub-chronic and chronic stages include fibrosis, immune responses, and inflammatory responses. Notably, cell cycle-related pathways are highly enriched in 401 genes that were differentially expressed only at 1 M.

**Figure 2:**
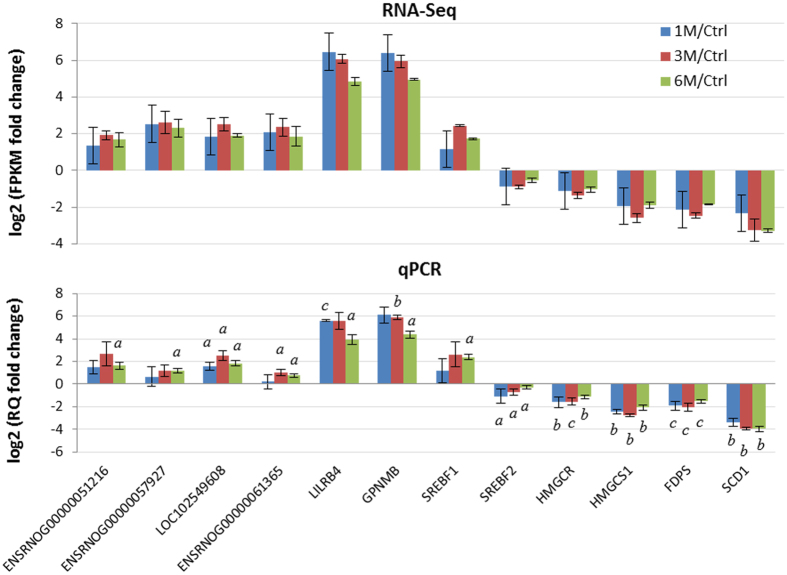
Verification of differential expression of selected genes by qPCR. Upper panel: Log2 FPKM fold-change in expression analyzed by RNA-Seq, calculated as the ratio of average FPKM of each time point to the control. Lower panel: Relative log2 of relative quantitation (RQ) fold-change in gene expression determined by qPCR, calculated using the 2^−ΔΔCt^ method. a: *p* < 0.05, b: *p* < 0.01, c: *p* < 0.001, compared to control using Student’s *t*-test. Data were shown as mean ± SD (n = 3).

Examining genes with the highest fold-change at each time point can provide insights into the major pathological events at different stages of SCI. Some of the top 10 DEGs (Supplementary Table S6) are well annotated in SCI or other neurological disorders, while others, including the lncRNAs, are less studied and require further investigation. Matrix metalloproteinase-12 (*Mmp12*), an extracellular matrix enzyme involved in blood-brain barrier disruption and CNS repair^16^, was significantly upregulated from 1 M through 6 M. Expression of *Scd1*, which encodes an enzyme controlled by sterol regulatory element binding proteins (SREBPs), was significantly decreased at all three time points examined. Interestingly, other genes that are regulated by SREBP in a similar manner and involved in myelination^17^, including *Hmgcr, Hmgcs1*, and *Fdps*, together with genes coding for SREBPs, were also significantly downregulated at multiple time points. Therefore we selected a subset of the top 10 DEGs (*Gpnmb, Lilrb4*, and *Scd1*) and aforementioned *Scd1*-associated genes for qPCR validation of differential expression. The fold-change in expression indicated by qPCR is very similar to the FPKM fold-change determined by RNA-Seq (Fig. 2).

### Identification of enriched gene sets by clustering analysis

Hierarchical clustering was performed to obtain an overview of the temporal expression dynamics of DEGs with fold-change (FC) > 2 and FDR < 0.01. The analysis resulted in five clusters among 4,910 genes (4,633 protein-coding and 277 lncRNA) (Fig. 1C; Supplementary Table S7). Cluster 1 consists of 790 protein-coding genes and 79 lncRNAs whose expression displayed gradual downregulation from control to sub-chronic and chronic stages. Enriched gene sets in this cluster are related to the neuronal system (synapse, synaptic transmission, and axon), myelin sheath, and cholesterol biosynthesis, and are likely downregulated due to neural cell death and demyelination that occur upon SCI. Cluster 2 consists of 540 protein-coding genes and 30 lncRNAs that exhibited significant upregulation at 3 M and reached their highest degree of upregulation at 6 M, and is enriched mostly in genes involved in cellular motility. The expression of Cluster 3, which includes 880 protein-coding genes and 46 lncRNAs, displayed gradual upregulation over time. Genes in this cluster are enriched in cell-to-cell interaction pathways including extracellular matrix, focal adhesion, and wound healing. The expression of Cluster 4, consisting of 1,420 protein-coding genes and 60 lncRNAs, reached its highest level of upregulation at 1 M, and then gradually returned to the baseline levels. Genes in cluster 4 are highly enriched for epigenetic modification mechanisms such as histone methylation, chromatin modification, methyltransferase activity, and RNA processing. Cluster 5 comprises 1,003 protein-coding genes and 62 lncRNAs that were robustly upregulated at 1 M and remained moderately upregulated at 3 M and 6 M. Cluster 5 is mainly enriched for genes involved in immune and inflammatory responses.

### Characteristics of lncRNAs in the rat genome

#### Distribution of lncRNA loci

**Figure 3:**
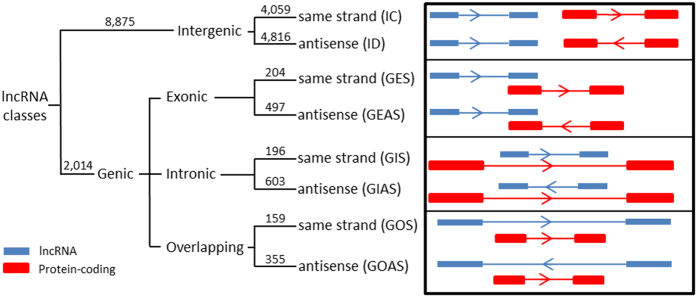
Classification of rat lncRNAs. Annotated rat lncRNAs were classified based on their genomic locations relative to protein-coding genes (see method section for more details). Number of lncRNAs in each class and subclass is indicated in parenthesis.

We categorized rat lncRNAs according to the location of their genes with respect to the most proximal protein-coding genes, based on a previous classification^18^ with modifications (Fig. 3). The lncRNA genes were first defined as ‘genic’ if they intersect a protein-coding gene, or ‘intergenic’ if they do not. Intergenic lncRNAs were further classified as ‘convergent’ (IC) if transcribed from the same strand or divergent (ID) if transcribed from the opposite strand. Genic lncRNAs were further subdivided into ‘genic exonic’, ‘genic intronic’, or ‘overlapping’, depending on whether they overlap with the exons or introns of a protein-coding gene. Exonic lncRNA genes, which overlap with at least one exon of a protein-coding gene, were categorized as GES if they lie on the same strand as the coding gene or as GEAS if they lie on the opposite strand. Intronic lncRNA genes, which overlap with the intronic region of a protein-coding gene, were further categorized as GIS if they lie on the same strand as the intron of a coding gene, or otherwise called GIAS. If a protein-coding gene is located completely within the intronic region of a lncRNA gene, this lncRNA is categorized as ‘overlapping’, and further classified as GOS if transcribed from the same strand as the coding gene, or GOAS if transcribed from the opposite strand.

Based on the combined lncRNA annotations, the majority of rat lncRNAs (8,875 out of 10,889 or 81.5%) were intergenic (37% IC and 44% ID). The remaining 2,014 lncRNA genes were genic lncRNAs [6.4% exonic (GES 1.9%, GEAS 4.5%), 7.3% intronic (GIS 1.8%, GIAS 5.5%), and 4.8% overlapping (GOS 1.5%, GOAS 3.3%), Fig. 3]. A complete list of annotated lncRNAs, including genomic locations and classifications is available in Supplementary Table S8.

#### ID lncRNAs are located closer to protein-coding genes than are IC lncRNAs

**Figure 4:**
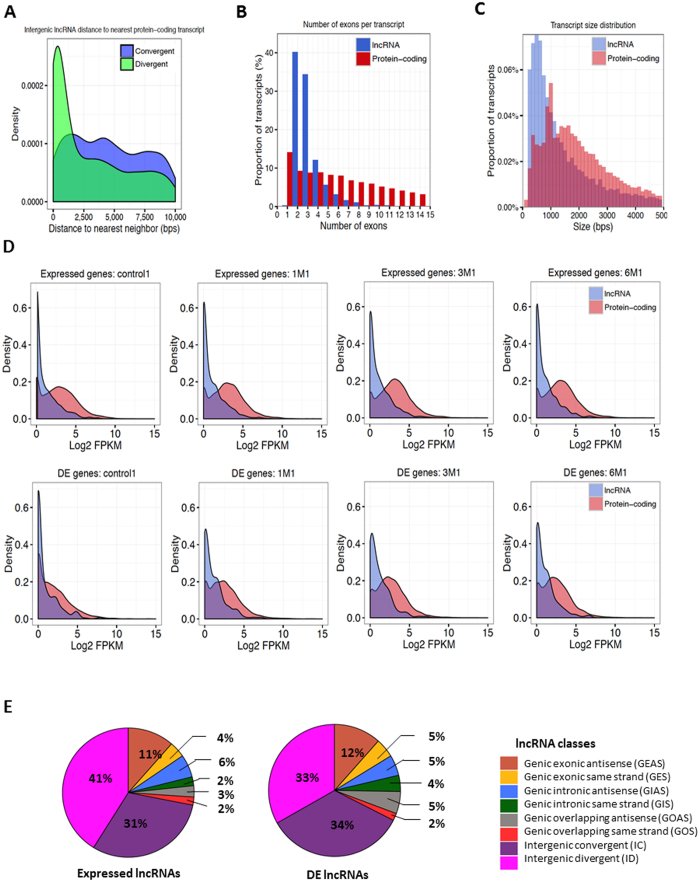
Characteristics of lncRNAs in the rat genome. (**A**) Distribution of the distances from IC lncRNAs (blue) or ID lncRNAs (green) to the closest protein-coding gene. Approximately 21.6% (1,039 out of 4,816) of ID transcripts and 10.4% (424 out of 4,059) of IC transcripts lie within 5 kb of a protein-coding gene (median distance 641 bp compared with 2,235 bp, respectively; *t*-test *p* < 1.41^−29^). (**B**) Number of exons per transcript for lncRNAs (blue) and protein-coding genes (red). The percentage of transcripts with only two exons is 40% and 7.5% for lncRNA and protein-coding transcripts, respectively. (**C**) Distribution of the transcript size of lncRNAs (blue) and protein-coding genes (red). The lncRNA transcripts are shorter than those of protein-coding genes (median transcript size 1,065 bp compared with 1,810 bp, respectively; *t*-test, *p* < 1.23^−145^). (**D**) Distribution of the FPKM values for DE lncRNAs (blue) and protein-coding genes (red) in control, 1 M, 3 M, and 6 M. Expressed and DE protein-coding genes displayed higher FPKM values in SCI samples when compared to control samples (1 M, 3 M, and 6 M compared with control for the expressed protein-coding genes: *p* < 8.17^−19^, *p* < 1.1^−42^, and *p* < 7.07^−26^; for DE protein-coding genes: *p* < 3.47^−47^, *p* < 1.34^−92^, and *p* < 3.14^−62^, respectively). (**E**) A pie chart depicting the distribution of each class of rat lncRNA (left: all expressed lncRNAs; right: DE lncRNAs post-SCI).

Distribution of the distances from intergenic lncRNAs to their nearest protein-coding gene neighbors showed that ID lncRNAs are closer to their neighbors than are IC lncRNAs. Approximately 21.6% (1,039 out of 4,816) of ID transcripts and 10.4% (424 out of 4,059) of IC transcripts lie within 5 kb of a protein-coding gene (median distance 641 bp compared with 2,235 bp, respectively, *t*-test *p* < 1.41^−29^, Fig. 4A).

#### lncRNAs exhibit specific exonic structure and transcript size compared to protein-coding genes

In our combined annotation there is a compelling tendency of lncRNA transcripts (40%) to have only two exons, whereas for protein-coding transcripts only 7.5% have two exons (Fig. 4B). These results are concordant with previous observations on human and rat lncRNAs^18^,^19^. The transcript size also differs between lncRNAs and protein-coding genes (Fig. 4C). Overall, lncRNA transcripts are shorter than protein-coding genes (median transcript size 1,065 bp compared with 1,810 bp; *t*-test, *p* < 1.23^−145^).

#### Differential expression of lncRNAs after SCI

After finding the DE protein-coding and lncRNA genes with DESeq, we compared their expression patterns at different time points using Kolmogorov-Smirnoff test. As illustrated in Fig. 4D, both expressed and DE lncRNAs generally had lower FPKM values compared to expressed and DE protein-coding genes across all time points (*p* < 2.2^−16^). Meanwhile, when comparing the FPKM distributions of all the expressed lncRNAs or DE lncRNAs of SCI samples to those of control samples, the expression levels are significantly higher in SCI samples (1 M, 3 M, and 6 M compared with control for all of the expressed lncRNAs: p < 0.0015, p < 4.88^−8^, and p < 1.8^−6^; and for DE lncRNAs: *p* < 3.94^−5^, 2.89^−10^, and 5.62^−8^, respectively). Similarly, the FPKM distributions of protein-coding genes are also significantly higher in SCI samples than control samples (for all of the expressed protein-coding genes, 1 M, 3 M, and 6 M compared with control: *p* < 8.17^−19^, *p* < 1.1^−42^, and *p* < 7.07^−26^; and for DE protein-coding genes: *p* < 3.47^−47^, *p* < 1.34^−92^, and *p* < 3.14^−62^, respectively). As observed in Fig. 4E, most of the 277 DE lncRNAs were classified as ID, IC, or GEAS. Same is true for the 1,035 expressed lncRNAs.

### Inferring potential functions of DE lncRNAs in rat SCI

**Figure 5:**
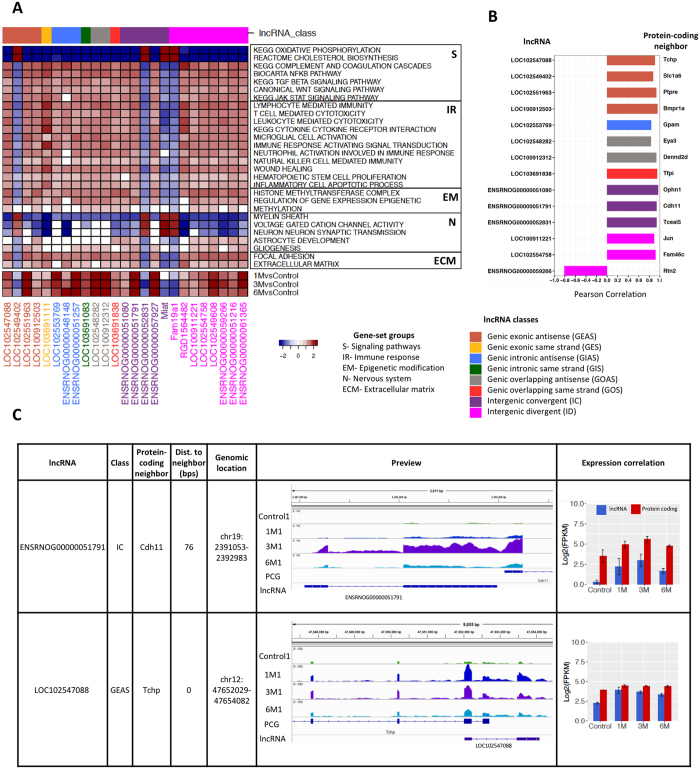
Inferring potential functions of DE lncRNAs in rat SCI. (**A**) Upper panel: Heatmap representing an association matrix of selected lncRNAs and enriched functional terms. Columns correspond to selected DE lncRNAs. Rows are enriched gene ontology terms and MsigDB canonical pathways. Enriched functional terms were categorized into signaling pathways (S), immune response (IR), epigenetic modification (EM), nervous system (N), and extracellular matrix (ECM). Color depth represents NES (normalized enrichment score) calculated by GSEA, indicating the association strength. Lower panel: Temporal expression assessed as log2(count fold-change) of selected DE lncRNAs. (**B**) Correlation coefficients between expression of DE lncRNAs and their closest protein-coding neighboring genes (see Methods). (**C**) Examples of DE lncRNAs and their closest protein-coding gene, with their classification and the distance to the nearest protein-coding gene neighbor indicated. The IGV browser view shows signal tracks for all time points. Bar plots indicate log2(FPKM) for protein-coding genes (red) and lncRNAs (blue). Error bars represent ± SD. PCG = protein-coding gene.

By correlating the expression profiles of DE lncRNAs with those of protein-coding genes, a ranked list of co-expressed protein-coding genes for each lncRNA was generated. Significantly enriched gene sets were identified using a Gene Set Enrichment Analysis (GSEA) with a false discovery rate (FDR) < 0.25. Figure 5A shows a list of selected DE lncRNAs with high normalized enrichment score (NES) based on the correlation of their expression with that of significantly enriched gene sets. The association matrix of significant gene sets and DE lncRNAs can be found in Supplementary Table S9. Enriched gene sets were classified into five categories including signaling pathways (S), immune response (IR), epigenetic modification (EM), nervous system (N), and extracellular matrix (ECM). A few representative DE lncRNAs in this list were selected for further validation by qPCR to verify their differential expression at multiple time points in the RNA-Seq data (Fig. 2).

The correlation between the expression of DE lncRNAs and that of their closest protein-coding genes was also investigated. All 277 DE lncRNAs were further filtered using two criteria: 1) the nearest protein-coding gene neighbor is also a DEG; and 2) the correlation of expression of the lncRNA and its neighboring protein-coding gene was significant (Pearson correlation, *p* < 0.05). This filtering yielded 77 lncRNAs including 16 GEAS, 2 GES, 3 GIAS, 2 GIS, 6 GOAS, 1 GOS, 22 IC, and 25 ID. Among these 77 lncRNAs, 51 (66%) are located within 5 kb of their significantly co-expressed protein-coding neighbor, 38 of which are transcribed from the antisense strand and 13 from the same strand. The expression of most of these neighboring DE lncRNA and protein-coding gene pairs was positively correlated (50 out of 51, 98%). Figure 5B shows representative lncRNAs and their significantly co-expressed protein-coding neighboring genes. A matrix of DE lncRNAs including lncRNA class, their closest protein-coding neighboring gene, the distance between them, and the correlation of their expression is available in Supplementary Table S10. Two example DE lncRNAs with expression significantly correlated to that of their DE protein-coding gene neighbors are depicted in further details (Fig. 5C). *ENSRNOG00000051791* is an IC lncRNA that is transcribed from the same strand as *Cdh11*, which codes for a cadherin membrane protein that mediates calcium-dependent cell-cell adhesion^20^. As shown in the genomic browser view, *ENSRNOG00000051791* and *Cdh11* are only 76 bp apart and display similar temporal changes in expression. *LOC102547088*, another DE lncRNA, is categorized as GEAS and overlaps with one exon of *Tchp*, a protein-coding gene with the ability to inhibit cell growth or act as a pro-apoptotic agent during cell stress^21^.

### TF binding motifs enriched in the regulatory regions of DE lncRNAs

**Figure 6:**
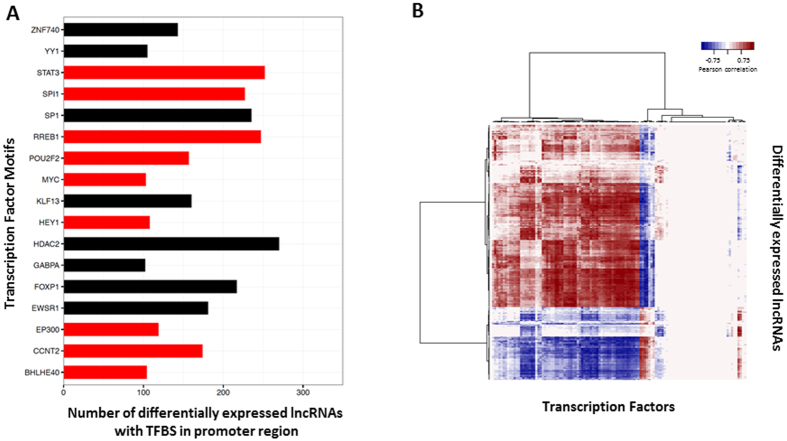
TF motifs found in the upstream regulatory regions of DE lncRNAs. (**A**) TFs with binding motifs found in more than 100 DE lncRNA regulatory regions. The *x*-axis indicates the number of DE lncRNAs containing binding motifs for particular TFs. DE TFs are shown in red and non-DE TFs in black. (**B**) Expression correlation matrix between DE lncRNAs and TFs with at least one motif in the upstream regulatory regions of DE lncRNAs. The matrix is colored-coded by Pearson correlation coefficients: color depth represents the correlation strength; red indicates positive correlation and blue indicatives negative correlation. The numerical correlation matrix is included in Supplementary Table S11.

The regulatory regions of DE lncRNAs (5 kb upstream and 1 kb downstream regions) were searched for TF binding motifs using FIMO (FDR < 0.05)^22^ with position weight matrices (PWM) obtained from ENCODE^23^, and a total of 200 motifs for 117 TFs were found. This list was further filtered to obtain TF motifs found in more than 100 lncRNA regulatory regions (Fig. 6A). Interestingly, 53% (9 out of 17) of these TFs are in our list of DEGs in SCI, including STAT3, RREB1, and SPI1. An association matrix depicting the presence of TF motifs in the regulatory regions of DE lncRNAs can be found in Supplementary Table S11.

To predict whether TFs with binding motif(s) in lncRNA regulatory regions are likely to regulate the lncRNAs, the correlations between the expression of the 117 TFs and that of the 277 DE lncRNAs were calculated. About 38.7% (12,563 out of 32,409) of the possible pairs are significantly correlated in their expression (Pearson coefficient *r* > |0.75|, *p*-value < 0.01), among which 71% are positively correlated and 29% are negatively correlated (Fig. 6B). Refer to Supplementary Table S11 for correlation coefficients between expression of TFs and that of DE lncRNAs.

### Rat lncRNAs homologous to disease-SNPs-harboring human genomic regions

An increasing number of studies are associating lncRNAs with human diseases^24^. Recently, genome-wide association studies (GWAS) have compiled information on a large number of disease-associated SNPs^25^. Notably, the majority of these SNPs were mapped to non-coding regions^26^. We lifted over rat SCI-associated DE lncRNAs to human genomic regions (hg38), yielding 224 homologous regions. Next, the GWAS SNP annotations were combined with the ClinVar genomic variation database to search for GWAS/ClinVar SNPs entries annotated in the aforementioned regions. We identified 23 rat DE lncRNAs that are homologous to human genomic regions that harbor disease-associated SNPs. For example, SNPs related to psychosis and Alzheimer’s disease were found in the human genomic region homologous to *LOC103693573*. Similarly, *LOC102555675* was homologous to regions harboring important SNPs involved in bipolar disorder in humans. The list of SNPs found in human homologs of rat DE lncRNAs is shown in Supplementary Table S12, including the rat DE lncRNAs, the genomic locations of their human homologs, the SNPs identified in GWAS/ClinVar, the trait or disease, the mapped human gene, and the reference PubMed ID.

## Discussion

Following mechanical and physical trauma caused by a contusive impact on the spinal cord tissue, a series of pathophysiological events occur at molecular and cellular levels. It is essential to understand how these events are orchestrated at a systemic level. The data presented here revealed critical pathways and networks that exhibit sustained alterations at the sub-chronic and chronic stages of SCI such as fibrosis and inflammatory responses, as well as pointed to the temporal regulation of important molecular and cellular machinery that potentially contribute to inhibition of axonal regeneration such as astrogliosis and compromised remyelination. The results of our analysis agree with recent findings by weighted gene coexpression network analysis (WGCNA) on microarray data from mouse spinal cord crush injury^27^ and a Bayesian network analysis (BNA) of RNA-Seq data from rat spinal cord transection injury^28^. Furthermore, our thorough annotations of rat lncRNAs, extensive analyses of DE lncRNAs to predict their potential regulatory functions in SCI, and identification of human disease-associated variants homologous to rat SCI-associated lncRNAs provided valuable resources for future functional investigations.

A major barrier to the treatment of chronic SCI is the increased astrogliosis that inhibits axonal growth and regeneration^29^. Our data demonstrated that genes related to gliosis are among the highly upregulated DE genes in the sub-chronic and chronic phases of SCI. Expression of CSPGs (*Ncan, Smc3*, and *Ptprz1*), Tenascin C (*Tnc*), *Slit3*^30^, and reactive astrocyte marker *Gfap* increased at all time points, and expression of Ephrin-B2 (*Efnb2*) increased at 3 M. Additionally, among the canonical pathways generated from DEGs that overlap in 1 M, 3 M, and 6 M, ‘fibrosis’ ranked as the most enriched. Upregulation of genes that code for collagen IV, laminin, and nidogen in our data confirmed the excessive synthesis of ECM components as a scaffold of basement membrane. The expression of multiple genes in the critical fibrosis signaling pathways TGFβ, WNT, and YAP/TAZ^31^, including *Tgfb1, Smad-2, 3, & 4, Wnt, Yap*, and *Ctnnb1*, increased from 1 M through 6 M. Many genes involved in fibrosis are also associated with glial scar formation. For example, *Egr1* (early growth response 1), a gene that was upregulated at three time points, encodes a TF that can regulate *Ptprz1* expression^32^ and modulate pathological matrix remodeling by enhancing collagen accumulation^33^.

**Figure 7:**
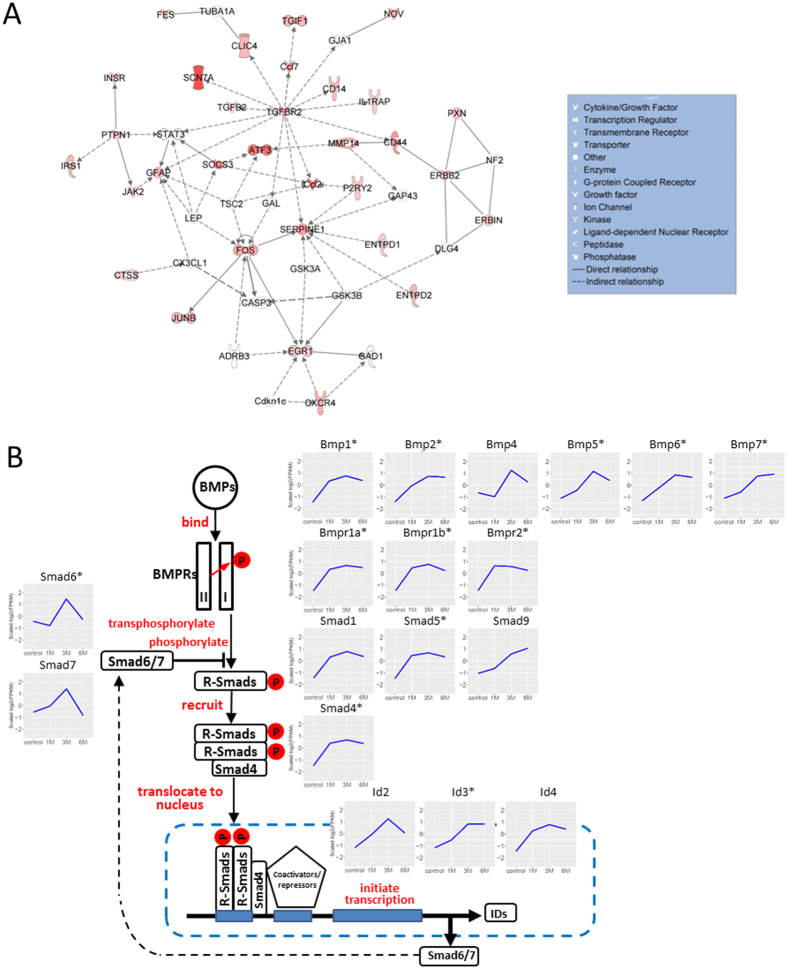
Enriched network and canonical pathway generated with DEGs that are important in chronic SCI. (**A**) One of the most enriched networks generated in IPA with 1 M/3 M/6 M overlapping DEGs. (*p*-score = 14 or *p*-value = 10^−14^). DEGs were colored-coded, with shades of red indicating the level of upregulation. *Stat3* was differentially expressed at 1 M and 3 M, after which it was marginally upregulated at 6 M (FC = 1.97). (**B**) A schematic illustration of gene expression profiles (log2-transformed FPKM) of members of the canonical Bone Morphogenetic Protein (BMP) pathway^73^ at different time points post-SCI. Genes coding for BMP ligands (BMP1, 2, 4, 5, 6 and 7), BMP receptors (BMPR1A, BMPR1B and BMPR2), SMADS (SMAD1, 5 and 9), transcriptional activator (SMAD4), and IDs (ID2, 3 and 4) exhibited upregulation in chronic SCI. Downstream target genes (such as *Olig1* and *Olig2*) showed a gradual downregulation pattern. Genes coding for inhibitory SMADS (SMAD6 and 7) displayed a transient upregulation peaking at 3 M and returning to basal levels at 6 M. *Indicates differentially expressed genes.

Network analysis provided further insights in molecular signaling that regulates gliosis after the sub-chronic and chronic SCI. We analyzed DE gene set common to 1 M, 3 M, and 6 M using Ingenuity Pathway Analysis (IPA) to generate networks depicting direct physical interactions such as binding and phosphorylation as well as indirect relationships inferred from literature in the Ingenuity Knowledge Base. In one of the most enriched networks (Fig. 7A), molecules with the greatest number of interactions include TGFBR2, GFAP, STAT3, and EGR1, among others, many of which are involved in astrogliosis and fibrosis. Transforming growth factor β receptor 2 (TGFBR2), which showed a 7–9-fold increase in gene expression from 1 M to 6 M post-SCI, had the most connections in the network, suggesting its important roles in gliosis. This is consistent with previous studies which suggest its ligand, TGF-β, functions as an essential regulatory factor of astrocyte function^34^ and gliosis by inducing synthesis of chondroitin sulfate proteoglycans^35^.

The expression of bone morphogenetic proteins (BMPs)^36^ and their receptors, which are members of the TGF-β superfamily, have been previously reported to increase 2 days after SCI and lasted for 1 month^37^. RNA-Seq data in the present study extends these findings to show that increased expression of BMP ligands (*Bmp1, 2, 4, 5, 6*, and *7*) as well as BMP receptors (*Bmpr1a, 1b*, and *2*) persists during chronic SCI at 3 M and 6 M post injury. BMP ligands bind to a complex of BMP receptors to initiate the transphosphorylation of receptor 1 which phosphorylates the receptor-activated Smads (R-Smads, Smad1, 5, and 9). The phosphorylated R-Smads form complexes with the co-Smad (Smad4) and move into the nucleus, where they combine with various transcriptional co-activators or co-repressors to regulate the transcription of target genes such as the inhibitors of differentiation (ID2, 3, and 4)^38^,^39^. Consistent with the activation of BMP pathways, our study shows not only increased expression of BMPs and their receptors but also upregulation of R-Smads and co-Smad as well as their target genes such as *ID2, 3*, and *4*. Interestingly, our study also shows upregulation of inhibitory Smads (*I-Smads, Smad6*/*7*) at 3 M. I-Smads are critical BMP responsive genes^40^,^41^,^42^. Expression of I-Smads, which are induced by BMP canonical pathway could prevent the further activation of R-Smads and inhibit BMP signaling. Thus, R-Smad/co-Smad and I-Smad form a negative feedback loop for the fine regulation of BMP signaling (Fig. 7B). At 6 M after SCI, BMP canonical pathway genes remain upregulated while I-Smads become significantly downregulated. Overall, these data indicate that BMP signaling is significantly increased in chronic SCI. Previous studies have implied that the BMP pathway can increase the proliferation and differentiation of astrocytes at the expense of oligodendrogenesis during development^43^ and after SCI^44^,^45^. The persistent upregulation of BMP signaling might be involved in maintaining the glial scar after chronic SCI. Moreover, BMP signaling also plays an important role in regulating angiogenesis during embryonic and postnatal development^46^. Therefore, our finding of sustained upregulation of the BMP pathway in chronic SCI requires further studies to achieve a greater understanding on the long-term effects of SCI on blood vessel integrity and replacement.

Another highly connected molecule in Fig. 7A, signal transducer STAT3, is connected indirectly to TGFBR2 and directly to GFAP. STAT3 has been proposed as a critical regulator of astrogliosis and axonal growth. Knockout of STAT3 in astrocytes resulted in decreased astrocyte reactivation and astroglial scar formation^47^, whereas activation of STAT3 in neurons was reported to enhance the axonal regeneration, especially in the initial phase of regeneration after injuries^48^. Interestingly, activation of STAT3 can upregulate the gene expression of *Socs3*, which can in turn inhibit STAT3 activity and impede axonal sprouting/regeneration^49^, possibly serving as a negative feedback to STAT3 activation to fine-tune its regulation in astrogliosis and axon regrowth after injury. In fact, both *Stat3* and *Socs3* were upregulated from 1 M through 3 M and *Socs3* stayed upregulated at 6 M. It is important to note that the transcriptomic alterations of the DEGs reported in our study represent changes in a mixture of cell types in the spinal cord tissue. Therefore, future studies such as co-localization with cell specific markers and classic genetic manipulation will help us to understand the functions and the underlying mechanisms of STAT3 and SOCS3 in axonal regeneration and astrogliosis. Additionally, SOCS3 is connected with transcription factor ATF3 which has been found to be induced in sensory and motor neurons after SCI^50^. ATF3 binds DNA sites as a homo- or hetero- dimer with JUN^50^. Interestingly, our analysis showed that both STAT3 and JUN are differentially expressed, have high number of binding motifs in the regulatory regions of DE lncRNAs, and have significant expression correlation with a subset of DE lncRNAs (Fig. 6). We hypothesize that these transcription factors may be involved in the upstream regulatory functions of lncRNAs in SCI.

To better understand the alterations in gene expression after SCI and their functional significance, it is essential to comprehend the transcriptional regulation of the genes involved. Systematic characterization and functional prediction of lncRNAs will significantly facilitate our understanding of transcriptional regulation. We created a combined annotation of 10,889 lncRNA transcripts derived from ENSEMBL and NCBI repositories. The majority of lncRNA annotations are predictions based on the methylation state of chromatin regions and the evaluation of coding potential. Our analyses showed that even though the genomic structure of lncRNAs is similar to that of protein-coding genes, lncRNAs are predominantly two-exon transcripts with a shorter median transcript length. Based on the current annotation databases, approximately two times as many intergenic divergent lncRNAs are located within less than 5 kb of their protein-coding neighbors as are intergenic convergent lncRNAs.

Our temporal analyses of the SCI transcriptome identified 277 DE lncRNAs in the sub-chronic and chronic stages of SCI. For example, *Miat* is a DE lncRNA that has been studied previously in other biological contexts. *Miat* is related to neuron commitment, development, and survival^51^, as well as oligodendrocyte lineage specification of neural stem cells^8^. Our results showed a downregulation of *Miat* expression after SCI, which could be due to neuronal death. Further investigations are necessary to validate this hypothesis. We would like to point out that in the present study, polyadenylated RNAs were selected using the polyA-based RNA-Seq library kit; therefore, lncRNAs that were not polyadenylated were not captured.

Through guilt-by-association methods, we were able to infer that DE lncRNAs are associated with functions related to signaling cascades, epigenetic modification, immune responses, nervous system, and extracellular matrix, all of which are highly relevant to SCI. To unravel the potential functional relevance of the proximity of lncRNAs to nearby protein-coding genes, we identified 51 pairs of DE lncRNAs and protein-coding gene neighbors that either overlap or lie within 5 kb and have statistically significantly correlated expression (Pearson correlation, *p*-value < 0.01). This finding suggests a role for these lncRNAs in *cis*-regulation of nearby protein-coding genes^52^.

To understand the upstream regulatory mechanisms of DE lncRNAs, we investigated the TF binding motifs present in their regulatory regions. Our results demonstrated that binding motifs for TFs relevant to gliosis (STAT3) and immune response (JUN^53^ and SP1^54^) are highly represented in the regulatory regions of DE lncRNAs, and that changes in the expression of these TFs are highly correlated with a subset of DE lncRNAs.

In summary, a high level of transcriptomic disturbance persisted in the sub-chronic and chronic phases of rat SCI. Our systematic examination and analysis of post-SCI transcriptional alterations in rat has identified important pathways and networks for the pathological progression of SCI, and pinpointed novel target genes for further investigation, including a number of interesting lncRNA candidates with potentially important regulatory functions and human disease homologs.

## Methods

All methods were carried out in accordance with relevant guidelines and regulations. All experimental protocols were approved by the Institutional Biosafety Committee at the University of Texas Health Science Center at Houston. Animal usage and manipulations were performed in accordance with the Public Health Service Policy on Humane Care and Use of Laboratory Animals, Guide for the Care and Use of Laboratory Animals, and with the approval of the Animal Welfare Committee at the University of Texas Health Science Center at Houston.

### Rat SCI model

A total of 36 female Sprague-Dawley rats (12–14 weeks of age) were used. Tissues from three rats were pooled to form one biological replicate (n). Animals were randomly assigned to either the control group or to one of the three time points (9 rats [3 biological replicates]): 1 M, 3 M, and 6 M. SCI surgeries were performed as described previously^55^. Briefly, a moderate (150 kdyn) contusive injury was induced to the 9^th^ thoracic vertebra (T9). Animals in the sham control group received a dorsal laminectomy without a contusive injury.

At 1 month, 3 months, and 6 months post-SCI, animals were intracardially perfused with 0.01 M PBS under anesthesia. A segment of the spinal cord tissue (0.5 mm) at the epicenter was dissected and snap-frozen. Animals in the control group were sacrificed at 6 months post-SCI and a section of the spinal cord tissue at an equivalent location was collected.

### RNA isolation, library construction, and RNA sequencing

Total RNA was extracted from the spinal cord tissue using TRIzol reagent (Invitrogen) following the manufacturer’s instructions. RNA quality was determined using a Bioanalyzer (Agilent), and all RNA integrity numbers (RINs) were greater than 8. About 150–300 ng total RNA was used to construct each RNA-Seq library. RNA samples were poly-A selected and paired-end sequencing libraries were constructed using the TruSeq RNA Sample Prep Kit, as described in the TruSeq RNA Sample Preparation V2 Guide (Illumina), and sequenced using the Illumina HiSeq 2000 sequencer. Library preparation and sequencing were performed in different batches based on the SCI time points and the sequencing availability. Batch assignments are included in Supplementary Table S1. Hierarchical clustering was performed to identify any possible batch effects.

### Read mapping and quantification

Before read mapping, the quality of raw sequenced reads was verified using FastQC^56^. Quality control metrics are listed in Supplementary Table S1. Read mapping, transcript assembly, and expression estimation were performed as described in our previous publication^14^. Reads were mapped to the rat reference genome Rnor6 downloaded from Ensembl (ftp://ftp.ncbi.nlm.nih.gov/genomes/all/GCF_000001895.5_Rnor_6.0/). The 100-bp paired-end reads were aligned to the reference genome using TopHat v2.1.0^57^ with default parameters. Mapped reads were assembled using Cufflinks v2.2.1^58^, and FPKM (Fragments Per Kilobase of transcript per Million mapped reads) values were obtained for genes and transcripts annotated. Our annotation included 39,183 transcripts spanning 30,443 genes (22,075 protein-coding and 8,368 lncRNA genes). Any FPKM < 0.1 was set to 0.1 to avoid ratio inflation^59^. Read counts for annotated genes and transcripts were obtained using HTSeq-count^60^.

### lncRNA annotation and categorization

We surveyed lncRNA public databases with known and predicted annotations. All gene and transcript biotypes labelled as ‘lincRNA’ from the Ensembl rat genome annotation were collected. Additionally, lncRNA annotations from NCBI repository (ftp://ftp.ncbi.nlm.nih.gov/genomes/all/GCF_000001895.5_Rnor_6.0/) were downloaded. The intersect function from Bedtools suite^61^ was used to avoid redundancy. Annotations were filtered by removing lncRNAs smaller than 200 nt and those with overlapping exons. The resulting lncRNA set consisted of 10,889 transcripts (2,901 from Ensembl and 7,988 from NCBI) spanning 8,370 loci. We categorized these lncRNAs according to their positions with respect to their closest protein-coding genes, based on previously published classification^18^ with modifications.

### Analysis of transcriptomic gene expression profiles and differential expression

The similarities between all samples were assessed using a consensus dendrogram generated from the Euclidean distances between log2-transformed quantile-normalized FPKM values of all samples. One outlier was identified in the 6 M group, so it was excluded from subsequent analyses. For more details on outlier identification, refer to “Sample correlation assessment” in Supplementary Methods.

Genes with FPKM 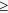
1 in at least one of the samples were included in the analysis of differential gene expression. Expression profiles of SCI samples at each time point were compared against those of the control group using the DESeq package^62^. Genes were classified as differentially expressed (DEGs) if they 1) had an FPKM > 1 in at least one sample, 2) exhibited an expression fold-change (FC) > 2 (mean normalized counts), and 3) their DESeq statistical test was significant with FDR < 0.01. Genes that met these three criteria were included in the downstream analyses.

### Pathway analysis

DEGs at each time point were imported into Ingenuity Pathway Analysis (IPA, http://www.ingenuity.com/products/ipa) to generate enriched pathways and networks. DEGs at each time point were also cross-compared to create lists of genes commonly expressed between time points or uniquely expressed at certain time points, as illustrated using Venn diagrams^63^ (Fig. 1B).

### Clustering temporal gene expression profiles

Hierarchical cluster analysis was performed using Ward’s method based on a Euclidean distance matrix of normalized log2-transformed quantile normalized FPKM values of DEGs. Genes within each cluster were used to estimate the enrichment of gene sets through a hypergeometric statistical test (phyper R function), downloaded from gene ontologies pre-built for rat^64^ and from the Molecular Signatures Database (MSigDB)^65^. Gene sets with an FDR < 0.05 were considered enriched. The number of clusters was selected so that the profiles of enriched gene sets showed the least redundancy. A heatmap was generated to depict these DEGs clusters and their corresponding enriched gene sets.

### Predicting potential functions of DE lncRNAs in SCI

We adopted a co-expression analysis of lncRNA genes and protein-coding genes to infer the potential functions of lncRNAs using a ‘guilt-by-association’ method^66^. The relationship between the expression of DE lncRNAs and protein-coding genes was assessed using Pearson correlation. A ranked list of protein-coding genes for each DE lncRNA was obtained. Gene Set Enrichment Analysis (GSEA)^67^ was used to identify significantly enriched gene sets corresponding to gene ontologies and canonical pathways from MSigDB^65^ for each lncRNA. Gene sets with an FDR < 0.25 (as recommended in the GSEA manual) were used to create an association matrix. A heatmap of a subset of DE lncRNAs was obtained showing the normalized enrichment score (NES) for selected significantly enriched gene sets.

### Correlation of expression of lncRNAs and protein-coding gene neighbors

To identify possible interactions between DE lncRNAs and their neighboring protein-coding genes, we first selected those pairs in which both the lncRNA and its protein-coding neighbor were differentially expressed and had a significant correlation (Pearson coefficient *r* > 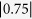, *p* < 0.01). We further filtered the list leaving only pairs that either overlap or are less than 5 kb apart. The Integrative Genomics Viewer (IGV)^68^ was used to browse the alignment files for DE lncRNAs and neighboring protein-coding genes.

### Identification of TF binding using ENCODE motifs

To identify TFs potentially involved in the regulation of DE lncRNAs, motif analysis was performed. The motif data set at ENCODE^23^ (http://compbio.mit.edu/encode-motifs) was downloaded. Motifs were used to scan the regulatory regions of DE lncRNAs using FIMO^22^ with an FDR < 0.05.

The regulatory regions of lncRNAs were defined as 5 kb upstream and 1 kb downstream from lncRNA transcription start sites. If any lncRNA was shorter than 1 kb, then its length was used as the cut-off value for the downstream region. Sequences were obtained using the BSgenome package^69^. A Pearson correlation matrix was computed for gene expression levels between TFs and lncRNAs with TF binding sites.

### Find rat lncRNAs homologous to disease-SNPs-harboring human genomic regions

To investigate the functional relevance of DE lncRNAs from our study to human diseases, we downloaded a collection of Genome-Wide Association Studies (GWAS)^70^ and Clinically Relevant Sequence Variations (ClinVar)^71^ to obtain a database of single-nucleotide polymorphisms (SNPs) linked to human diseases and traits. We lifted-over the DE lncRNAs from rat (Rnor6) to human (hg38) genome using the UCSC liftOver Utility^72^. A table was constructed of DE lncRNAs in rat SCI, their homologous regions in human, and the diseases or traits linked to these regions (Supplementary Table S12).

### Reverse transcription, quantitative PCR (qPCR) verification, and statistical analysis

The differential expression of selected protein-coding genes and lncRNAs was validated by qPCR. Complementary DNAs (cDNAs) were synthesized from the same RNA samples as those used for the RNA-Seq experiment using SuperScript II (Invitrogen #18064–014) and random primers following the manufacturer’s instructions. SYBR^®^ Green (Bio-Rad #172–5122) was used as detector in qPCR reactions. Primer sequences are available in Supplementary Table S13. GAPDH was used as an endogenous control. The 2^-ΔΔCt^ method was used for relative quantification of expression.

Student’s *t*-tests were performed to compare gene expression at each time point to that of control, and significance was established when *p* < 0.05.

### Data availability.

(1) Raw RNA-Seq datasets have been deposited in Gene Expression Omnibus (GEO) database under accession number GSE93249.

(2) Sequence alignments and lncRNA annotations are publicly available to the research community for browsing (http://jiaqianwulab.org/ratSCI/ratSCI.html).

## Additional Information

**How to cite this article**: Cuevas-Diaz Duran, R. *et al*. The systematic analysis of coding and long non-coding RNAs in the sub-chronic and chronic stages of spinal cord injury. *Sci. Rep.* **7**, 41008; doi: 10.1038/srep41008 (2017).

**Publisher's note:** Springer Nature remains neutral with regard to jurisdictional claims in published maps and institutional affiliations.

## Supplementary Material

Supplementary Information

Supplementary Table S1

Supplementary Table S2

Supplementary Table S3

Supplementary Table S4

Supplementary Table S5

Supplementary Table S6

Supplementary Table S7

Supplementary Table S8

Supplementary Table S9

Supplementary Table S10

Supplementary Table S11

Supplementary Table S12

Supplementary Table S13

## Figures and Tables

**Table 1:** Summary of differentially expressed genes (DEGs) in rat SCI.

Category		Total DEGs	Up in SCI	Down in SCI
Protein-coding genes	1 M	2,935	2,805	130
	3 M	3,981	3,265	716
	6 M	3,206	2,545	661
Long non-coding (lncRNAs)	1 M	137	120	17
	3 M	239	162	77
	6 M	179	125	54

The numbers of DEGs relative to control are shown for each time point. Criteria for inclusion of DEGs: FPKM values > 1 in at least one sample, fold-change (FC) > 2, and FDR < 0.01.
