# Supplementary material for: The systematic analysis of coding and long non-coding RNAs in the sub-chronic and chronic stages of spinal cord injury
Source: Sci Rep. 2017 Jan 20;7:41008. doi: 10.1038/srep41008 (PMC5247719; doi:10.1038/srep41008)
Supplement: Supplementary Information [file srep41008-s1.doc]

**The systematic analysis of coding and long non-coding RNAs in the sub-chronic and chronic stages of spinal cord injury**

Raquel Cuevas-Diaz Duran^#,1,2^, Han Yan^#,1,2^, Yiyan Zheng^1,2^, Xingfan Huang^3^, Raymond Grill^4,&^, Dong H. Kim^1^, Qilin Cao^1,2^*, and Jia Qian Wu^1,2^*

^#^ Contributed equally to the work.

*Co-corresponding authors.

**SUPPLEMENTARY METHODS**

**Animal and SCI surgery**

Female Sprague-Dawley rats (12–14 weeks of age, body weight 220-250g) were purchased from Charles River Laboratories. Under general anesthesia (ketamine 60 mg/kg and xylazine 7 mg/kg, i.p.) and aseptic conditions, a T9 dorsal laminectomy was performed to expose the dorsal surface of the T9 spinal cord segment. The spine was stabilized using steel stabilizers inserted under the transverse processes one vertebra above (T8) and below (T10) the injury site. All animals except the sham control received a moderate T9 contusive injury with a 150 kdyne force and 500 ms dwell time delivered with an Infinite Horizon (IH) Spinal Cord Impactor device (Infinite Horizon’s, LLC, Lexington, KY). Core temperature was maintained at 37°C during surgery with a homeothermic blanket. Immediately after the injury, the musculature and skin incision were sutured with sterile 5-0 Vicryl and 2 mL lactated Ringer’s solution was given subcutaneously. Animal in the sham control group received laminectomy without contusion. Each animal was given 0.25% bupivicaine solution subcutaneously at 2.5mg/kg as analgesic right before surgery. After surgery, each animal received subcutaneous injection of buprenorphine at 0.05 mg/kg twice per day for 3d. Gentamycin was injected subcutaneously at 2 mg/kg once right before surgery and then daily for 3d post-injury. Bladders were emptied twice per day for 7d post-injury or until voiding returned. Rats that were not hind limb-paralyzed 24hr post-injury were excluded from the study, which indicated a failed induction of moderate contusive spinal cord injury. Rats that could not reach water or food, had severe infection in the paralyzed limb(s), or exhibited signs of unrelieved pain, autophagy, or excessive weight loss which persists for 2 weeks were euthanized and excluded.

**Sample processing and quality control**

Library preparation and sequencing of samples were performed in different batches based on the SCI time points and the sequencing availability. Batch assignments are listed in Supplementary Table S1. No batch effects were identified. After sequencing, sample quality was assessed by metrics obtained using FastQC^1^. The most relevant metrics for all the sample are listed in Supplementary Table S1.

**Sample correlation assessment**

The similarity among biological replicates was determined by the pairwise Pearson correlation coefficient of log2 transformed and quantile normalized FPKM values of expressed genes among all samples. The correlation matrix included in Supplementary Table S1 shows that the similarity among the biological replicates is high (greater than 96%); however, the correlation coefficient is lower between sample 6M3 and other replicates from 6M. Therefore, we did not include this outlier sample in the final analyses.

**SUPPLEMENTARY FILES**

**Supplementary Table S1.** Contains information about the processing/sequencing batches, sample quality control metrics, and sample correlation coefficients. Quality control metrics were computed with FastQC. Pearson correlation coefficients were calculated pairwise from the log2 quantile normalized FPKM values of all the expressed genes.

**Supplementary Table S2.** Protein-coding and lncRNA gene expression levels (FPKM) calculated using Cufflinks v2.2.1 with default parameters. Any FPKM less than 0.1 was set to 0.1 to avoid ratio inflation. The column ‘type’ is used to differentiate protein-coding genes from lncRNAs.

**Supplementary Table S3.** Differential expression analysis results obtained comparing SCI samples (1M, 3M, and 6M) to sham controls. Analysis was performed with DESeq using the raw counts for all annotated genes. For each time point, genes are divided into protein-coding genes and lncRNAs (separate worksheets in XLS file). The column “expressed” indicates if the gene’s FPKM > 1 in at least one sample. The column “upRegIn” indicates if the gene was considered differentially expressed and upregulated in SCI or control group. Differentially expressed genes fulfilled three criteria: a) are expressed, b) FC >2, and c) FDR < 0.01. Differentially expressed genes DEGs common to all time points or unique to each time point are listed as separate worksheets.

**Supplementary Table S4.** Changes in gene expression of cell-specific markers ^2,3,4^ for astrocytes, neurons, oligodendrocytes, microglia/macrophages, and endothelia. Changes in gene expression represent the alterations in the spinal cord tissue which consists of a mixture of different types of cells. Mean log2 of fold-changes in DESeq normalized read counts at each time point represent the fluctuations of expression. Values for DEGs indicate significant upregulation (red) or downregulation (green), and non-DEGs were labeled as ‘NS’.

**Supplementary Table S5.** Canonical pathways enriched at each time point after SCI. Only significantly enriched pathways (*p*-value < 0.05) are listed, with each time point shown on a separate worksheet. Positive z-score indicates a canonical pathway is activated and negative z-score indicates a canonical pathway is inhibited, while ‘N/A’ indicates that prediction for the pathway is not currently possible. More detailed information regarding the analysis and parameters can be found at: [http://www.ingenuity.com/products/ipa#/?tab=resources](http://www.ingenuity.com/products/ipa#/?tab=resources).

**Supplementary Table S6.** Top 10 DEGs (including protein-coding genes and lncRNAs) from each time point according to the log2 fold-changes in DESeq mean normalized read counts.

**Supplementary Table S7.**  Hierarchical cluster membership of DE protein-coding genes and lncRNAs (fold-change > 2 and FDR < 0.01). Related to heatmap in Fig. 1C.

**Supplementary Table S8.** Combined lncRNA annotation obtained from Ensembl and NCBI repositories. File includes gene ID, transcript ID, genomic location, status, and locus classification (related to Fig. 3). The “status” column indicates if the gene annotation is known (matching a public scientific database such as RefSeq) or novel (predicted or computationally derived).

**Supplementary Table S9.** Association matrix of DE lncRNAs and functional gene sets. Gene sets (rows) and lncRNAs (columns) are associated with NES (normalized enrichment score) value calculated using GSEA. Only associations with FDR < 0.25 are presented in the matrix. Related to Fig. 5A.

**Supplementary Table S10.** DE lncRNA classification. DE lncRNAs are listed with their closest protein-coding gene neighbor, the distance between them, Pearson correlation coefficients for expression, and a column (both.DE) depicting whether both genes (lncRNA and protein-coding neighbor) are differentially expressed. Related to Figs. 5B and C.

**Supplementary Table S11.** Predicted TF binding motifs. The tab ‘TF.motifs’ contains an association matrix between DE lncRNAs (rows) and TFs (columns) with TF motifs present in the regulatory regions of more than 100 lncRNAs. A value of ‘1’ indicates that a TF binding motif was found, ‘0’ indicates that a known TF binding motif was not found. The tab ‘correlation.TF.DE.lncRNA’ corresponds to the Pearson correlation coefficients obtained by comparing log2-transformed FPKM expression between DE lncRNAs and TFs throughout all samples. Related to Figs. 6A and B.

**Supplementary Table S12.** Rat DE lncRNAs located in homologous regions harboring SNPs in the human genome.

**Supplementary Table S13.** Primer sets for qPCR verification.

**References**

1. FastQC: A quality control tool for high throughput sequence data. Available at: http://www.bioinformatics.babraham.ac.uk/projects/fastqc/. (Accessed: 1st January 2016)

2. Zhang, Y. *et al.* An RNA-Sequencing Transcriptome and Splicing Database of Glia, Neurons, and Vascular Cells of the Cerebral Cortex. *J. Neurosci.* **34,** 11929–47 (2014).

3. Darmanis, S. *et al.* A survey of human brain transcriptome diversity at the single cell level. *Proc. Natl. Acad. Sci.* **112,** 7285–7290 (2015).

4. Kozlenkov, A. *et al.* Differences in DNA methylation between human neuronal and glial cells are concentrated in enhancers and non-CpG sites. *Nucleic Acids Res.* **42,** 109–27 (2014).
